# The inoculum effect of antibiotics against CTX-M-extended-spectrum β-lactamase-producing *Escherichia coli*

**DOI:** 10.1186/s12941-014-0045-1

**Published:** 2014-09-12

**Authors:** Na Wu, Bai Yi Chen, Su Fei Tian, Yun Zhuo Chu

**Affiliations:** Division of Infectious Disease, The First Affiliated Hospital, China Medical University, Shenyang, China; Department of Laboratory Medicine, The First Affiliated Hospital, China Medical University, Shenyang, China

**Keywords:** Extended-spectrum β-lactamases (ESBLs), Inoculum effect, *Escherichia coli*

## Abstract

**Background:**

Questions remain regarding the use of the cephalosporins to treat infections caused by extended-spectrum β-lactamase (ESBL)-producing *Escherichia coli*. For example, should ceftazidime or cefepime be used to treat infections with CTX-M ESBL-producing organisms with low MICs (minimum inhibitory concentrations), according to the new Clinical and Laboratory Standards Institute’s (CLSI) recommendations for susceptibility testing? Some studies have reported that in vitro MICs of cephalosporins increase as the inoculum increases, which is the inoculum effect; however, most of the enzymes studied were SHV and TEM. In this study, we aimed to investigate the inoculum effect on ceftazidime, cefepime and four other β–lactam agents against CTX-M-ESBLs-producing *Escherichia coli*.

**Methods:**

Antibiotic susceptibilities were determined using broth microdilution MIC methodology according to the CLSI recommended with standard and 100-fold-higher inocula.

**Results:**

An inoculum effect on meropenem and cefminox was not detected. The size of the inoculum affected piperacillin/tazobactam activity against only 4 strains, all CTX-M-14 genotypes. The inoculum size affected the activity of ceftazidime, cefepime and cefotaxime against 35%, 85%, 100% of strains, respectively. Among the strains with an inoculum effect, CTX-M-14 was the most common ESBL genotype.

**Conclusions:**

These findings suggest that meropenem is the most active compound against serious infections caused by *Escherichia coli* producing ESBLs. Cefminox and piperacillin-tazobactam exhibit strong activity against many strains. Until further studies are performed, clinicians should be aware that third- and fourth-generation cephalosporins (such as ceftazidime and cefepime) are not reliable for serious infections even though in vitro tests indicate susceptibility.

## Introduction

The CLSI has revised susceptibility breakpoints for *Enterobacteriaceae* and recommendations for testing for ESBL production, and now recommends reporting the MICs of cephalosporins, but not the production of ESBLs [1]. Consequently, many ESBL-producing *Escherichia coli* may be reported susceptible to ceftazidime or cefepime, especially those producing CTX-M-ESBL, which are apt to hydrolyze cefotaxime [2]. However, clinical correlations that support the effectiveness of these agents against ESBL-producing organisms infections are lacking. Moreover, some in vitro studies show poor outcomes using ceftazidime or cefepime to treat the serious infections with ESBL-producing organisms [3,4]. One reason for this disparity is the inoculum effect, which means the MICs of cephalosporins increase as the inoculum increase. Some in vitro studies have shown an inoculum effect against SHV- or TEM-ESBL-producing isolates [5,6]. It is unknown whether the inoculum effect extends to the CTX-M genotypes, the most widespread type of ESBLs in Asia, especially in China [7,8]. Therefore, we aimed to investigate the inoculum effect on ceftazidime, cefepime and other four kinds of β–lactam agents against CTX-M- ESBL-producing *Escherichia coli*.

## Methods

Eighty non-replicate strains of *Escherichia coli*, identified using the Vitek 2 system (bioMérieux S.A., Marcy I^’^Etoile, France), were investigated in this study. The isolates were selected from a collection of clinical isolates of patients from the First Hospital of China Medical University. ESBL production was confirmed phenotypically using double-disc diffusion tests with ceftazidime with/without clavulanic acid and cefotaxime with/without clavulanic acid. β-lactamases produced by isolates were characterized by PCR gene sequencing, including CTX-M-14, CTX-M-15, CTX-M-22, CTX-M-24 and CTX-M-79 (as reported by our previous study [9]). *Escherichia coli* ATCC25922 was used as the quality control strain for susceptibility testing. This study was approved by the Ethics Committee of our hospital (Approval number, 2013114) and conducted in accordance with the ethical guidelines of the Declaration of Helsinki.

MICs were determined by the broth microdilution method in accordance with CLSI (2010) recommendations [1]. Susceptibility testing was performed using inoculum concentrations of approximately 1–5 × 10^5^ CFU/ml (the standard inoculum) and 1–5 × 10^7^ CFU/ml (the higher inoculum). Inoculum concentrations were estimated by optical density measurement and verified by quantitative subculture. An inoculum effect was defined as an eightfold or greater increase in MIC when tested with the high inoculum [5]. Comparision betweent CTX-M-14-group and other-genotype-group were performed by chi-square test using SPSS16.0. P value of < 0.05 were considered statistically significant. Antimicrobial agents meropenem, cefminox, piperacillin/tazobactam (tazobactam 4 μg/ml), cefepime, ceftazidime and cefotaxime (obtained from the National Institute for the Control of Pharmaceutical and Biological Products) were tested.

## Results

The strains were isolated from blood (n = 40), urine (n = 25), ascites (n = 7), bile (n = 5) and pus (n = 3). The sequence analysis of lactamase-producing organisms detected the ESBL genotypes CTX-M-14 (n = 44), CTX-M-15 (n = 8), CTX-M-22 (n = 12), CTX-M-24 (n = 4) and CTX-M-79 (n = 12). At the standard inoculum, the MICs of cefotaxime were higher than the MICs of cefepime and ceftazidime against all CTX-M strains (Table 1). The MICs of meropenem and cefminox were very low and less affected by the inoculum. An inoculum effect on piperacillin/tazobactam was observed in only 4 strains, all genotype CTX-M-14. The MIC_50_ of ceftazidime was 4 μg/ml at the low inoculum and 35% (28/80) of strains showed an inoculum effect at the high inoculum (all 28 strains were genotype CTX-M-14). An inoculum effect on cefepime was observed for 85% (68/80) of isolates, the frequencies of this inoculum effect, by genotype were CTX-M-14, 40/44; CTX-M-15, 8/8; CTX-M-22, 12/12; CTX-M-24, 4/4; and CTX-M-79, 4/12.

**Table 1:** **The inoculum effects on six antimicrobial agents for 80 strains of** ***Escherichia coli*** **at standard and high**-**inoculum**

**Antimicrobial agent**	**MIC** **(μg/** **ml)** **at standard and high inoculum**						**Number of strains showing an inoculum effect**	***Escherichia coli*** **ATCC25922**
	**Standard inoculum**			**High inoculum**				
	**(1**–**5 × 10** ^**5**^ **CFU/** **ml inoculum)**			**(1**–**5 × 10** ^**7**^ **CFU/** **ml inoculum)**				
	Range	MIC50	MIC90	Range	MIC50	MIC90		
Meropenem	≤0.015 ~ 0.06	0.03	0.03	0.03 ~ 0.06	0.03	0.06	0	≤0.015
Cefminox	0.5 ~ 4	1	2	0.5 ~ 8	2	4	0	2
Piperacillin/tazobactam	4 ~ 32	8	32	8 ~ 256	8	64	4	2
Ceftazidime	1 ~ 32	4	16	8 ~ 128	32	64	28	0.25
Cefepime	4 ~ 32	8	32	32~ > 512	512	>512	68	0.12
Cefotaxime	16 ~ 256	64	256	128~ > 512	512	>512	80	0.12

## Discussion

The production of ESBLs is the predominant cause of resistance to β-lactam antibiotics in gram-negative bacteria. However, the antimicrobial substrate specificities of different phenotypes of ESBLs vary. The common phenotypes of the ESBL enzymes are TEM, SHV, CTX-M and others. More recently, the CTX-M β-lactamases, which have potent hydrolytic activity against cefotaxime, have been the most widespread β-lactamases in Asia, especially in China. Therefore, we studied strains containing CTX-M-encoded genes to explore their presumptive role as a cause of therapeutic failure. In this study, the isolates contained CTX-M-14, CTX-M-22, CTX-M-15, CTX-M-24 and CTX-M-79 encoding genes. CTX-M-14 is the most common genotype in our area. CTX-M-79 was first reported by Su Fei Tian et al. in our previous study [9].

In this study of CTX-M-ESBL-producing isolates, the inoculum effect on cefotaxime susceptibility tests was found to be most frequent; it was observed in 100% of strains. The elevated cefotaxime MICs might be explained by the potent hydrolytic activity of CTX-M-ESBLs against cefotaxime and could be the underlying cause of therapeutic failure.

Based on the antimicrobial susceptibility data, most CTX-M-14 strains were susceptible to ceftazidime at the standard inoculum. However, at the high inoculum we found the inoculum effect on ceftazidime was frequent, MICs of 28 (35%) strains increased dramatically using the high inoculum. Although the MICs of ceftazidime were often low, ceftazidime might not be effective against severe infections (such as abscesses, endocarditis, meningitis, septic arthritis, osteomyelitis, and other deep-seated infections with high concentrations of bacteria) due to the inoculum effect. All isolates with this inoculum effect contained the CTX-M-14 encoding gene, which is the most common type in our area. In our area, we should scrutinize the use of ceftazidime to treat such infections. Isolates containing the newly reported genotype, CTX-M-79, did not show this inoculum effect.

There is disagreement about the use of cefepime to treat infections caused by ESBLs-producing organisms when the in vitro testing indicates susceptibility. Cefepime is reported to be less prone to hydrolysis by ESBLs [10] than other cephalosporins. Notably, in animal models of infections with such organisms, treatment with cefepime has produced both successful and unsuccessful therapeutic outcomes. In this study, at the standard inoculum, the MIC_50_ of cefepime was 8 μg/ml. Using the high inoculum, MICs of most isolates (68/80) increased more than eight-fold, showing an inoculum effect. We examined the genotypes of strains with this inoculum effect: as Table 2 shows, 40 strains had CTX-M-14,and 28 strains belonged to other types (CTX-M-15, 8; CTX-M-22, 12; CTX-M-24, 4; and CTX-M-79, 4). Making comparision betweent CTX-M-14-group and other-genotype-group by *χ*^2^ test, the two groups displayed significant variousity (P = 0.001). Considering the widespread prevalence of CTX-M-14-ESBL-producing organisms and this inoculum effect, cefepime may be a less reliable agent for therapy of serious ESBLs infection.

**Table 2:** **Analysis of strains with an inoculum effect on cefepime**

**Strain group**	**Number of strains showing inoculum effect**	**MIC**_**90**_ **(μg/** **ml)**	
		**Standard inoculum** **(5** ** × ** **10** ^**5**^ **CFU/** **ml)**	**High inoculum** **(5** ** × ** **10** ^**7**^ **CFU/** **ml)**
CTX-M-14 producing strains	40^a^	16	>512
Other genotypes	28	16	>512

^a^P = 0.001 by chi-square test.

Piperacillin-tazobactam was less subject to an inoculum effect, it was only observed in four strains of CTX-M-14 derived ESBLs. Piperacillin-tazobactam might have better efficacy against pathogens that produce CTX-M enzymes. A recent series analyzed the outcome of 43 episodes of *Escherichia coli* bacteremia caused by ESBL-producing strains (primarily CTX-M-14). The mortality rate of patients given a β-lactam/β-lactamase inhibitor combination was lower than that of patients given either a cephalosporin or fluoroquinolone [11]. However, in the study of Lopez-Cerero et al., the inoculum effect of piperacillin-tazobactam was more frequent than we observed [12]. This difference may be due to differences in ESBL genotypes, which were primarily TEM- and SHV-ESBLs in Lopez-Cerero’s study, whereas our isolates were all CTX-M ESBLs. It also may be related to MICs differences among isolates from different regions. All need further investigation.

At the standard-inoculum and the high inoculum, the MICs of meropenem and cefminox were very low, with no inoculum effect. The finding of meropenem is consistent with previous reports of strains producing SHV- and TEM-derived ESBLs [5,6]. It suggests that carbapenems, such as meropenem, could be the best choice for treating infections caused by ESBL producers. Although we also detected no inoculum effect on cefminox, given the limited data about the use of cefminox to treat ESBL-producing organisms, further investigations are warranted to evaluate its clinical efficacy.

## Conclusions

In tests of CTX-M-ESBL-producing *Escherichia coli* isolates, meropenem and cefminox were less influenced by inoculum size. Piperacillin-tazobactam was subject to an inoculum effect in the presence of certain ESBLs. Inoculum effects were detected more frequently with cefepime, ceftazidime and cefotaxime. These findings suggest that meropenem could be the most active compound against serious infections caused by *Escherichia coli* producing ESBLs. Cefminox and piperacillin-tazobactam exhibited strong activity against many of the isolates. Until further studies are performed, clinicians should be aware that third- and fourth-generation cephalosporins (such as ceftazidime and cefepime) are not reliable for serious infections even though in *vitro tests* indicate susceptibility.

## Abbreviations

ESBLs: Extended-spectrum β-lactamases
CLSI: Clinical and Laboratory Standards Institute
MIC: Minimum inhibitory concentration
